# The Use of Social Media for Health Research Purposes: Scoping Review

**DOI:** 10.2196/25736

**Published:** 2021-05-27

**Authors:** Charline Bour, Adrian Ahne, Susanne Schmitz, Camille Perchoux, Coralie Dessenne, Guy Fagherazzi

**Affiliations:** 1 Department of Population Health Luxembourg Institute of Health Strassen Luxembourg; 2 Inserm U1018 Center for Research in Epidemiology and Population Health (CESP) Paris Saclay University Villejuif France; 3 Epiconcept Paris France; 4 Competence Centre for Methodology and Statistics Luxembourg Institute of Health Strassen Luxembourg; 5 Luxembourg Institute of Socio-Economic Research Esch/Alzette Luxembourg

**Keywords:** social media, public health, epidemiology, research, health, medical, social networking, infodemiology, eHealth, text mining

## Abstract

**Background:**

As social media are increasingly used worldwide, more and more scientists are relying on them for their health-related projects. However, social media features, methodologies, and ethical issues are unclear so far because, to our knowledge, there has been no overview of this relatively young field of research.

**Objective:**

This scoping review aimed to provide an evidence map of the different uses of social media for health research purposes, their fields of application, and their analysis methods.

**Methods:**

We followed the scoping review methodologies developed by Arksey and O’Malley and the Joanna Briggs Institute. After developing search strategies based on keywords (eg, social media, health research), comprehensive searches were conducted in the PubMed/MEDLINE and Web of Science databases. We limited the search strategies to documents written in English and published between January 1, 2005, and April 9, 2020. After removing duplicates, articles were screened at the title and abstract level and at the full text level by two independent reviewers. One reviewer extracted data, which were descriptively analyzed to map the available evidence.

**Results:**

After screening 1237 titles and abstracts and 407 full texts, 268 unique papers were included, dating from 2009 to 2020 with an average annual growth rate of 32.71% for the 2009-2019 period. Studies mainly came from the Americas (173/268, 64.6%, including 151 from the United States). Articles used machine learning or data mining techniques (60/268) to analyze the data, discussed opportunities and limitations of the use of social media for research (59/268), assessed the feasibility of recruitment strategies (45/268), or discussed ethical issues (16/268). Communicable (eg, influenza, 40/268) and then chronic (eg, cancer, 24/268) diseases were the two main areas of interest.

**Conclusions:**

Since their early days, social media have been recognized as resources with high potential for health research purposes, yet the field is still suffering from strong heterogeneity in the methodologies used, which prevents the research from being compared and generalized. For the field to be fully recognized as a valid, complementary approach to more traditional health research study designs, there is now a need for more guidance by types of applications of social media for health research, both from a methodological and an ethical perspective.

**International Registered Report Identifier (IRRID):**

RR2-10.1136/bmjopen-2020-040671

## Introduction

### Social Media Background

Social media (SM) refer to new forms of media that involve interactions between users [1] in personal (eg, Facebook) or more professional (eg, LinkedIn) ways. In 2010 in the United States, 80% of adults used the internet to search for health-related information, and 11% of SM users posted comments, queries, or information about health or medical content [2]. Every user activity on the internet generates a unique digital footprint that can be collected for health research [3]. However, SM are not only used in a personal way. Indeed, academics are also increasingly using SM to share their work and disseminate their findings [4].

### Opportunities for Health Research

Since the creation of SM in 2004-2005 and with 3.81 billion active social media users in April 2020 [5], concepts like infodemiology and infoveillance have emerged. The term “infodemiology” refers to the science of using the internet to improve public health, while “infoveillance” refers to the science of syndromic surveillance using the internet [6]. These opportunities have been seized through the years in order to create new methodologies for health research to cope with the issues raised by traditional methods (eg, difficulty of recruitment [7]).

### Scoping Review Contextualization

Previous scoping and systematic reviews have already been published about the different uses of SM for health research. However, they were either focusing on a specific type of SM (eg, blogs [8]), on a specific field of health research (eg, child maltreatment [9]), or on a specific methodology (eg, recruitment of study participants [10,11]). Other reviews discussed the overall use of SM for health research [12,13] but did not provide any insights on the analysis techniques or the ethical issues. Besides, the COVID-19 pandemic has sped things up and pushed research to be done online, leveraging existing data for disease surveillance purposes, which makes the present work particularly timely and needed for better structuration of the field [14]. The research field on social media and health is relatively young and therefore lacks structures and guidelines. In the light of the above, it seemed important to map the different uses of social media for health research. Our work will directly contribute to the general effort of acknowledging the potential of this research field and will help to identify the main limitations to tackle in the future.

### Review Questions

The overall research questions were as follows: (1) How have SM modified or complemented traditional health research? (2) What are the different fields of application of this approach? (3) What are the different methodologies for SM data analysis?

## Methods

### Overview

This scoping review followed the methodological framework introduced by Arskey and O’Malley in 2005 [15] and the methodology manual published by the Joanna Briggs Institute for scoping reviews [16]. It is reported in accordance with the Preferred Reporting Items for Systematic Reviews and Meta-analyses Extension for Scoping Review (PRISMA-ScR) guidelines [17]. The methods have been previously detailed in a research protocol [18].

### Search Strategy

An initial literature search was first manually conducted on PubMed/MEDLINE to identify the health research fields in which SM are mostly used and developed. We searched for the term “social media” in the Medical Subject Headings (MeSH) Terms (words or phrases selected to represent particular biomedical concepts) as it gathers all papers discussing the use of at least one example of social media. For instance, this MeSH Term also includes articles that mention Facebook or Twitter without referring directly to “social media.” We considered the term “health research” as all kinds of research performed to learn more about human health, prevent or treat disease, test ideas, improve treatments, and answer questions. Then, the literature search was performed through PubMed/MEDLINE and Web of Science. The search strategy, highlighted in Textbox 1, included two sets of search terms: (1) one linked with SM (eg, social media) and (2) one linked with research (eg, health research, biomedical research). In order to capture the evolution of SM uses for health research over the years, databases were searched between January 1, 2005, and April 9, 2020. The term “social network” was also searched, as it is often misused as a synonym of SM. An additional list of 5 relevant articles [19-23] was manually searched to identify any other potentially relevant articles not yet captured. These articles were chosen in order to retrieve more articles about infodemiology, ethical issues, or the use of SM data. A snowball searching technique was adopted with these 5 articles in which citations within articles were searched and kept if relevant to the review.

**Inclusion criteria, exclusion criteria, and search strings.**

**Inclusion criteria**

- written in English
- published between January 1, 2005, and April 9, 2020
- dealt with the use of social media by researchers

**Exclusion criteria**

- not about health research
- not related to social media (eg, social network analysis)
- not about human subjects
- no relevant information (eg, methodology) about the use of social media for health research
- no relevant characteristics of social media

**Search string in PubMed**

(((“Social Media”[MH]) OR (“Social Media”[TW])) AND ((“Biomedical research”[MH]) OR (“Medical research”[TW] OR “Biomedical research”[TW]) OR (“Health research”[TW] OR “Health services research”[TW]))) OR (((“Social networking”[MH]) OR (“Social network”[TW] OR “Social networks”[TW] OR “Social networking”[TW])) AND ((“Biomedical research”[MH]) OR (“Medical research”[TW] OR “Biomedical research”[TW]) OR (“Health research”[TW] OR “Health services research”[TW]))) Filters: Journal Article; Publication date from 2005/01/01 to 2020/04/09; Humans; English

**Search string in Web of Science**

(TS=“Social Media” OR TS= “Social networking” OR TS= “Social network” OR TS= “Social networks”) AND (TS=“Biomedical research” OR TS=“Medical research” OR TS=“Health research” OR TS=“Health services research”) AND (PY=(2005-2020)) AND (LANGUAGE: (English)) Indexes=SCI-EXPANDED, SSCI, A&HCI, CPCI-S, CPCI-SSH, ESCI Timespan=All years

### Eligibility Criteria

This review was guided by the “Population, Concept, Context” framework suggested by the Joanna Briggs Institute [24]. We did not have any restriction about the population; we took any relevant publications regardless of the age, the origin, or the gender of the studied populations. The concept was the use of social media and the context was health research. The eligibility criteria were any journal article that described the use of social media platforms or social media data for health or medical research purposes. We excluded articles that were not directly related to research from our review, such as those on the use of social media among patients, patient associations or communities, organizations, or health care professionals for their day-to-day practice. Grey literature and studies about nonhuman subjects were excluded as well. Documents related to the mining of social media data to detect prescription drug misuse and abuse as well as those related to the use of machine learning methodologies to analyze data were eligible for inclusion. We included full texts that reported on at least one of the following outcomes: (1) SM data analysis, (2) recruitment through SM, (3) methodology for SM research, and (4) ethical issues of using SM for health research. Only English-language articles were retained. The inclusion and exclusion criteria and the search strings are summarized in Textbox 1.

### Study Selection Process

A 2-step screening was performed after duplicate removal. First, titles and abstracts were screened in order to define the eligibility of each article. Publications with title or abstract not meeting the eligibility criteria were excluded. Then, the full texts having passed the first step were screened, and only articles meeting the eligibility criteria were kept. All screening levels were conducted with CADIMA [25], a free web tool to facilitate the conduct and documentation of literature reviews [26]. Two reviewers screened articles (GF, CB) independently, and consistency checks were performed thanks to CADIMA.

### Data Extraction

Data were abstracted on (1) the country of origin, (2) the aims of the study (eg, to map ethical issues when using SM for health research), (3) the type of study (eg, recruitment feasibility assessment), (4) the research field (eg, mental health research), (5) the studied population (eg, adolescents), (6) the type of SM (eg, Facebook), (7) the methodology (eg, paid advertisement), (8) the outcomes of the study (eg, efficiency of recruitment via SM), and (9) the key findings for our scoping review (eg, possibility to recruit on SM). Data were extracted and cleaned by a first reviewer (CB), then verified and approved by a second reviewer (GF).

### Methodological Quality Appraisal

Because this is a scoping review, we did not appraise methodological quality or risk of bias of the included articles.

### Analysis and Presentation of Results

We conducted a descriptive analysis of the characteristics of the included literature. We described the included articles according to the journal of publication, publication date, country of origin (location of the corresponding author), Altmetric score (automatically calculated weighted count of all of the attention a research output has received) [27], type of SM, type of population, and type of disease studied. We decided to focus on Altmetric score rather than citation counts; as the SM research field is still relatively young, traditional citation counts provide a quite conservative approach of a paper’s “influence” that is influenced by the size of the research community working on the topic. Thus, Altmetric might be less influenced by the relatively “young” aspect of this research field by giving weight to other dimensions (record of dissemination, influence, impact). All these measures are more nuanced than citation counts alone are able to be [28,29]. However, Altmetric scores also have some limitations, as they do not take comparability across journals and platforms into account, and this system can be gamed [30,31].

We categorized the diseases in 7 categories: (1) chronic diseases (eg, diabetes), (2) communicable diseases (eg, influenza), (3) alcohol/smoking (eg, vaping), (4) mental health (eg, depression), (5) lifestyle (eg, nutrition outcomes), (6) drug/medication (eg, drug use disorder), and (7) other (eg, child maltreatment). Descriptive statistics and corresponding plots were computed (n, means, frequencies) with R (version 3.6.3; R Foundation for Statistical Computing).

## Results

### Search Results

**Figure 1:**
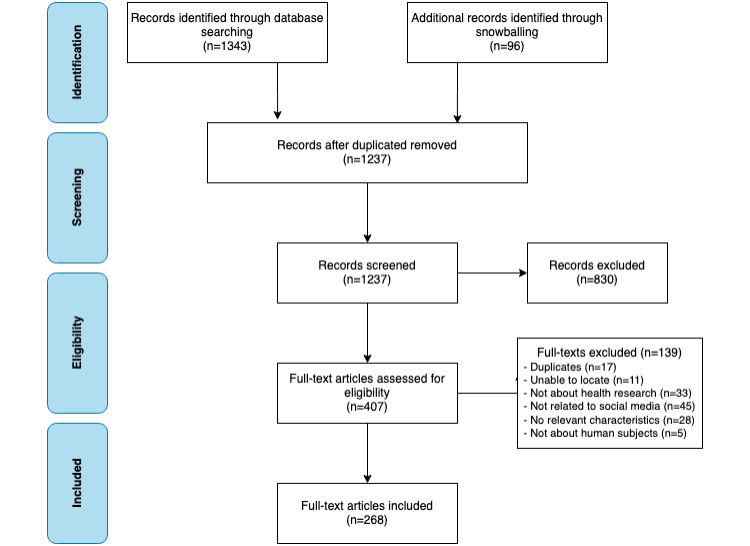
Flow diagram of the included studies.

The initial search conducted in April 2020 revealed 1343 results. An additional 96 articles were retrieved through a snowballing technique based on 5 relevant articles [16-20]. This resulted in a total of 1439 articles, and duplicates (n=202) were removed. Then, 1237 titles and abstracts were screened, which led to the exclusion of 830 articles. Overall, 407 studies were included to screen as full‐text papers, of which 139 were excluded. The main reasons for exclusion were that the study (1) did not contain relevant characteristics of SM for health research (n=28), (2) did not relate to SM (n=45), or (3) was not about health research (n=33). 268 studies were included in the analyses. Figure 1 shows the flow diagram of the article selection. Lastly, Multimedia Appendix 1 displays the characteristics of the 268 included studies (author or authors, year of publication, country, title, aim of the study, type of social media, studied population and disease).

### Distribution of Studies

In all, we included 268 unique records from 155 different journals. Table 1 displays the 10 most common journals in which the included studies were published: 55 (20.5%) articles were published in the *Journal of Medical Internet Research* or sister journals *JMIR Research Protocols* and *JMIR Public Health and Surveillance*. *PLoS ONE* is the second most common journal with 10 (3.7%) articles.

**Table 1:** Top 10 most common journals publishing work using social media for health research purposes.

Name of the journal	Articles, n (%)
Journal of Medical Internet Research	39 (14.6)
PLoS ONE	10 (3.7)
JMIR Research Protocols	9 (3.4)
JMIR Public Health and Surveillance	7 (2.6)
American Journal of Public Health	5 (1.9)
The American Journal of Bioethics	5 (1.9)
BMC Medical Informatics and Decision Making	4 (1.5)
International Journal of Environmental Research and Public Health	4 (1.5)
PLoS Computational Biology	4 (1.5)
Digital Health	3 (1.1)

A total of 1025 authors took part in the writing of the included studies. Figure S1 in Multimedia Appendix 2 provides the coauthorship network of all these authors. The largest set of connected authors included 57 authors and shown in Figure S2 in Multimedia Appendix 2).

Even though our research date range was from 2005 to 2020, none of the 268 included articles are dated before 2009. In Table 2, it can be seen that the number of publications is growing through the years, corresponding to an average annual growth rate of 32.7% for the 2009-2019 period. This suggests that the field of health research supplemented by SM has gained interest for the last 11 years. Earlier studies concentrated more on the use of SM for health research in general and the opportunities for the study of communicable diseases. The most recent studies more frequently included recruitment strategies and methodologies. Table 3 displays the distribution of articles by the continent of publication. Most articles were from the Americas (173/268, 64.6%, including 151/173, 87.3% from the United States), 18.7% were from Europe (50/268), 11.6% were from Oceania (31/268), 4.9% were from Asia (13/268), and 0.4% were from Africa (1/268).

**Table 2:** Distribution of publications by year of publication.

Year	Publications, n
2009	2
2010	3
2011	7
2012	5
2013	20
2014	26
2015	34
2016	37
2017	42
2018	36
2019	45
2020 (Jan 1–Apr 9)	11

**Table 3:** Distribution of publications by geographic location (as assessed by the location of the corresponding author).

Geographic location	Publications, n
Africa	1
The Americas	173
Asia	13
Europe	50
Oceania	31

### Social Media

Among all the retrieved articles, 57.8% (155/268) used or described at least one specific type of SM. From these articles, as can be seen in Table 4, 42.6% (66/155) were based on Twitter, 34.2% (53/155) on Facebook, and 11.0% (17/155) on several SM (eg, combining Facebook, Instagram, and Snapchat [32]). The remaining 12.3% (19/155) were distributed between Instagram, Reddit, forums, blogs, Weibo, and YouTube.

**Table 4:** Distribution of publications by social media (N=155).

Type of social media	Publications, n
Blogs	2
Facebook	53
Forums	3
Instagram	5
Reddit	5
Several types	17
Twitter	66
Weibo	2
YouTube	2

### Focused Populations

A total of 80.2% (215/268) of included articles did not focus on any specific population. In articles that studied a specific subpopulation (n=53), youth was the most common one (34/53, 64%), followed by women (7/53, 13%), families (5/53, 9%), men (1/53, 2%), and other (6/53, 11%), as shown in Table 5. The “Other” category gathered adults (2/6), Chinese migrants (1/6), elderly people (1/6), emergency nurses (1/6), and researchers (1/6).

**Table 5:** Distribution of publications per studied population (N=53).

Studied population	Publications, n
Youth	34
Women	7
Families	5
Men	1
Other	6

### Domain of Health Research

In addition, 45.1% (121/268) of publications dealt with a specific disease or condition (the remaining articles usually dealt with the use of SM for health research in general or with methodology). Indeed, as shown in Table 6, 33.1% (40/121) of articles studied communicable diseases, 19.8% (24/121) studied chronic diseases, 15.7% (19/121) studied lifestyle (eg, nutrition outcomes), 13.2% (16/121) studied other conditions (eg, drug use disorder), 9.9% (12/121) studied alcohol/smoking (eg, vaping), and 8.3% (10/121) studied mental health (eg, depression).

**Table 6:** Distribution of publications by studied disease type (N=121).

Studied disease type	Publications, n
Alcohol/smoking	12
Chronic diseases	24
Communicable diseases	40
Lifestyle	19
Mental health	10
Other	16

#### Communicable Diseases

Among articles that discussed communicable diseases, influenza was the primary studied disease (18/40, 45%), followed by HIV (8/40, 20%) and human papillomavirus (3/40, 8%).

#### Chronic Diseases

Among articles that discussed chronic diseases, a quarter studied cancer (6/24, 25%), followed by diabetes (5/24, 21%), cardiovascular diseases (eg, congenital heart disease, 3/24, 12%), and obesity (2/24, 8%).

### Dissemination

**Figure 2:**
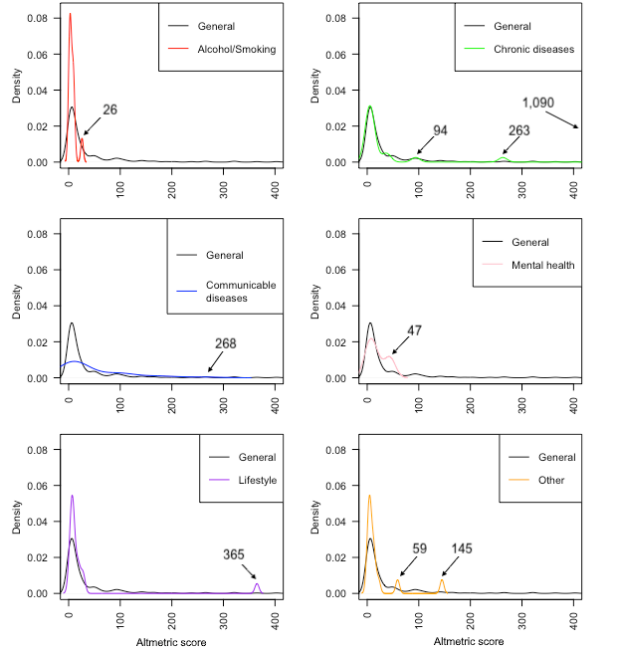
Distribution of Altmetric scores by health research area. Corresponding publications to the indicated Altmetric scores: Alcohol/Smoking: 26 [33]; Chronic diseases: 94 [34], 263 [35], 1090 [36]; Communicable diseases: 268 [37]; Mental health: 47 [38]; Lifestyle: 365 [39]; Other: 59 [40], 145 [41]. General corresponds to the Altmetric scores of all studies.

As highlighted in Figure 2, some papers stood out and could be considered important in the recent field of health research on social media.

### Type of Studies

Among all included studies, 22.4% (60/268) described the use of machine learning and data mining techniques, 22.0% (59/268) discussed the opportunities and limitations of the use of SM for research, 16.8% (45/268) assessed the feasibility of recruitment strategies on SM, 6.0% (16/268) discussed the ethical issues when using SM for health research, 5.2% (14/268) gave methodologies for health research, and 4.9% (13/268) illustrated the use of SM for dissemination. Guidelines for recruitment (9/268, 3.4%), interventions of prevention (6/268, 2.2%), crowdfunding (4/268, 1.5%), sentiment analysis (4/268, 1.5%), data anonymization (2/268, 0.7%), and crowdsourcing (2/268, 0.7%) were also considered.

### Machine Learning and Other Techniques

Machine learning techniques included text mining (17/60, 28%), natural language processing (15/60, 25%), data mining (12/60, 20%), classification (10/60, 17%), topic modelling (4/60, 7%), deep learning (1/60, 2%), and social network analysis (1/60, 2%). In particular, support vector machine (17/60, 28%), logistic regression (11/60, 18%), latent Dirichlet allocation (5/60, 8%), convolutional neural network (5/60, 8%), random forests (4/60, 7%), decision trees (4/60, 7%) and n-grams (64/60, 7%) are the most used models. Stacked linear regression, Bayesian network algorithm, nonnegative matrix factorization, stochastic gradient, learning vector quantization, and recurrent neural networks represent 2% (1/60) each. Lastly, these techniques were mostly used for data coming from Twitter (38/60, 63%) and Reddit (3/60, 5%).

### Recruitment Strategies

Studies assessing recruitment strategies’ feasibility applied paid advertisement (36/45, 80%), free advertisement (eg, posting in relevant Facebook groups [42]) (6/45, 13%), and the combination of both advertisements (3/45, 7%). Paid recruitment strategies included designing the ad, targeting the right audience with Facebook Ads Manager and measuring the impacts with Facebook Analytics [43]. Moreover, 64% (29/45) of studies considered SM recruitment to be effective (time-effective and efficient to recruit populations). Paid advertisement was evaluated as cost-effective in 83% (30/36) of studies and too costly in 6% (2/36). We found out that 80% (36/45) of recruitment was carried out on Facebook, 9% (4/45) on both Facebook and Twitter, and 9% (4/45) on more than two types of SM (eg, Facebook, Twitter, Craigslist, Tumblr, LinkedIn [44]). Lastly, a third of recruitment strategies included providing incentives to participants (eg, gift cards).

### Ethical Issues

Ethical issues were usually mentioned but not investigated in detail. When the article focused on ethical issues (n=16), the main ethical issues raised were getting consent of online users (15/16, 94%), protecting the privacy of users (14/16, 88%), preserving confidentiality (9/16, 56%), potential harms to participants (9/16, 56%), preserving of anonymity (8/16, 50%), securing data (7/16, 44%), transparency of the research (7/16, 44%), application of guidelines (7/16, 44%), representativeness and self-selection bias (5/16, 31%), and the risk of double accounts (2/16, 12%).

## Discussion

### Principal Findings

The overarching aim of this review was to scope the literature for evidence on the use of SM for health research. We were able to include 268 studies. Most of the included articles in this scoping review are dated from 2013 onwards, which is consistent with the worldwide growth of SM use over the last decade [45]. We identified three main SM used for health research: Twitter, Facebook, and Instagram, the most popular platforms in 2020 [46]. The most studied populations are young adults and adolescents. This could be related to the elevated proportion of young people active on SM. In 2018 in the United States, 51% of teens were on Facebook, 69% on Snapchat, 72% on Instagram, and 85% on YouTube; thus, SM seems to have great potential to focus on the young generations [47]. The majority of the included works focused on both communicable and chronic diseases. The field of SM research is still very young, and this can be seen in the impact that publications have (via the Altmetric score), with the exception of 9 articles. However, it is set to evolve rapidly, and it will be necessary to follow the evolution of the Altmetric scores of the field in the coming years to identify the new major articles.

The fields of application of SM in health research are broad and constantly evolving: as earlier studies concentrate on the study of communicable diseases, most recent studies include recruitment strategies and data collection for infoveillance. First, SM can be used to complement traditional methods. Traditional procedures can meet several limitations. When recruiting a specific population, traditional methods (eg, fliers, advertising) can be expensive or limited in reach [7,48-50]. Complementing them with SM advertisements can cope with these limitations. Second, SM alone show high potential. Studies have concluded that SM paid advertisements can be an efficient and cost-effective tool to recruit [11,51-56]. SM appear not only to facilitate and complement traditional recruitment strategies to reach specific populations but to be efficient as well when used alone [52,57-59], especially to reduce time constraints or to target a large population [60]. Particularly, Facebook can be used to recruit participants of all ages and allows researchers to obtain participant samples similarly representative to those recruited via traditional recruitment methods [11]. Facebook, together with Facebook Ads Manager and Facebook Analytics, are particularly useful to develop and adjust such strategies. Traditional disease surveillance, population surveillance, and epidemiology methodologies can be improved by SM [21,50,61]. Pharmacovigilance and the detection of adverse drug reactions on SM proved to be efficient and to reduce time between the online report of an incident and its discovery [62-64]. As the number of SM users is increasing, generated data, or “big data,” is expanding. Such data can be collected and studied to improve disease and public health surveillance [65-67] to forecast diseases [68] or to improve research in a medical field [69,70]. Along with big data growth, machine learning and data mining techniques such as text mining and natural language processing are constantly evolving and are thus increasingly used in the field of public health research based on SM [71-73]. These techniques can be particularly interesting to analyze social media data and, for instance, to develop sentiment or topic analysis among a specific population [19,74] or to predict epidemics [75]. Twitter is mainly used for such work because Twitter developed a streaming application programming interface. This is a free application that allows easy access to 1% of all Twitter data in real time, filtered by specific criteria (eg, keywords) [76,77]. Lastly, SM can be directly used by health researchers to support prevention interventions to raise awareness and engage populations [78] and to crowdfund by promoting their research on SM. Indeed, crowdfunding can be eased by establishing professional contacts through SM and sharing campaigns [79].

The digitization of public health and clinical research is likely to grow in the years to come. The COVID-19 pandemic has already played a major role in this dynamic. Indeed, social media were not only efficient to spread information and to share diagnostic, treatment, and even follow-up protocols [80-82] but also to develop infoveillance studies to help characterize disease distribution and behaviors critical to the early stages of an outbreak [83,84] and to recruit participants in order to collect large-scale data within a short time period [85].

Still, the use of SM features and SM data for health research induces several ethical issues and limitations. Online data, such as those from Twitter, are often considered to be public, and user consent is not provided for collecting it. Moreover, ensuring privacy protection of a data set when anyone has access to vast amounts of public information is difficult because data could be reidentified [86,87]. Safety features should be used to protect users’ personal and sensitive information [20] and to protect users from dangerous or fake content posted by detractors, chatbots, or social media trolls (people who purposely provoke other SM users) [88]. These kinds of behaviors can also be oriented to researchers themselves and demotivate them. Moreover, data can represent only certain users’ characteristics due to researchers’ self-selection or to coverage issues of underserved populations or minority groups who are disproportionately absent online (eg, older adults). This can bias the representativeness of the sample and consequently bias the findings and prevent from any generalizability [89,90]. However, it is possible to multiply platforms (cross-platforms) or to combine with other recruitment methods to minimize such bias [91]. When recruiting and providing incentives, users might be tempted to participate multiple times. Researchers should ensure that the study allows only one response from a given IP address [92,93]. A few guidelines and frameworks have already been created to guide health researchers in using social media and prevent such issues [94-98].

### Strengths and Limitations of This Scoping Review

The present work used a rigorous scoping review methodology from the manual by the Joanna Briggs Institute [16] throughout the entire process. It was guided by a previously published protocol [18]. To ensure a broad search of the literature, the search strategy included two electronic bibliographic databases and the snowball technique. There are some limitations to our scoping review process. We may not have identified all relevant articles in the published literature despite attempts to be as comprehensive as possible. We limited our review to documents written in English, which may have led to missed relevant studies. Data were abstracted by one reviewer and verified by a second reviewer because of the important number of included publications.

### Conclusion and Recommendations

Our findings suggest that SM hold high potential to improve and complement existing health research studies. Indeed, some SM features can complement traditional research strategies, and the growing amounts of SM data hold great opportunities in the evolution of infoveillance and infodemiology. For researchers, SM can be an effective tool at almost every step of a study, from the development, ideation, recruitment, and crowdsourcing to the dissemination of findings. Researchers should determine which type of SM best fits their objectives, as Facebook might be better for recruitment and Twitter for data collection, in order to gain time and efficiency. Last but not least, we have observed strong heterogeneity in the approaches used. We therefore recommend taking the existing guidelines into account and carefully thinking about the different ethical issues highlighted in this work before using SM for research.

## Appendix

Multimedia Appendix 1 Overview of the included studies by year, country of origin, aim, population, type of social media and disease. NR: not relevant.

Multimedia Appendix 2 Coauthorship network.

## Abbreviations

MeSH: Medical Subject Headings
PRISMA-ScR: Preferred Reporting Items for Systematic Reviews and Meta-analyses Extension for Scoping Reviews
SM: social media
