# Supplementary figures and images for: The Use of Social Media for Health Research Purposes: Scoping Review
Source: J Med Internet Res. 2021 May 27;23(5):e25736. doi: 10.2196/25736 (PMC8193478; doi:10.2196/25736)

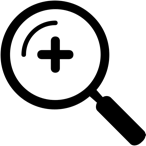

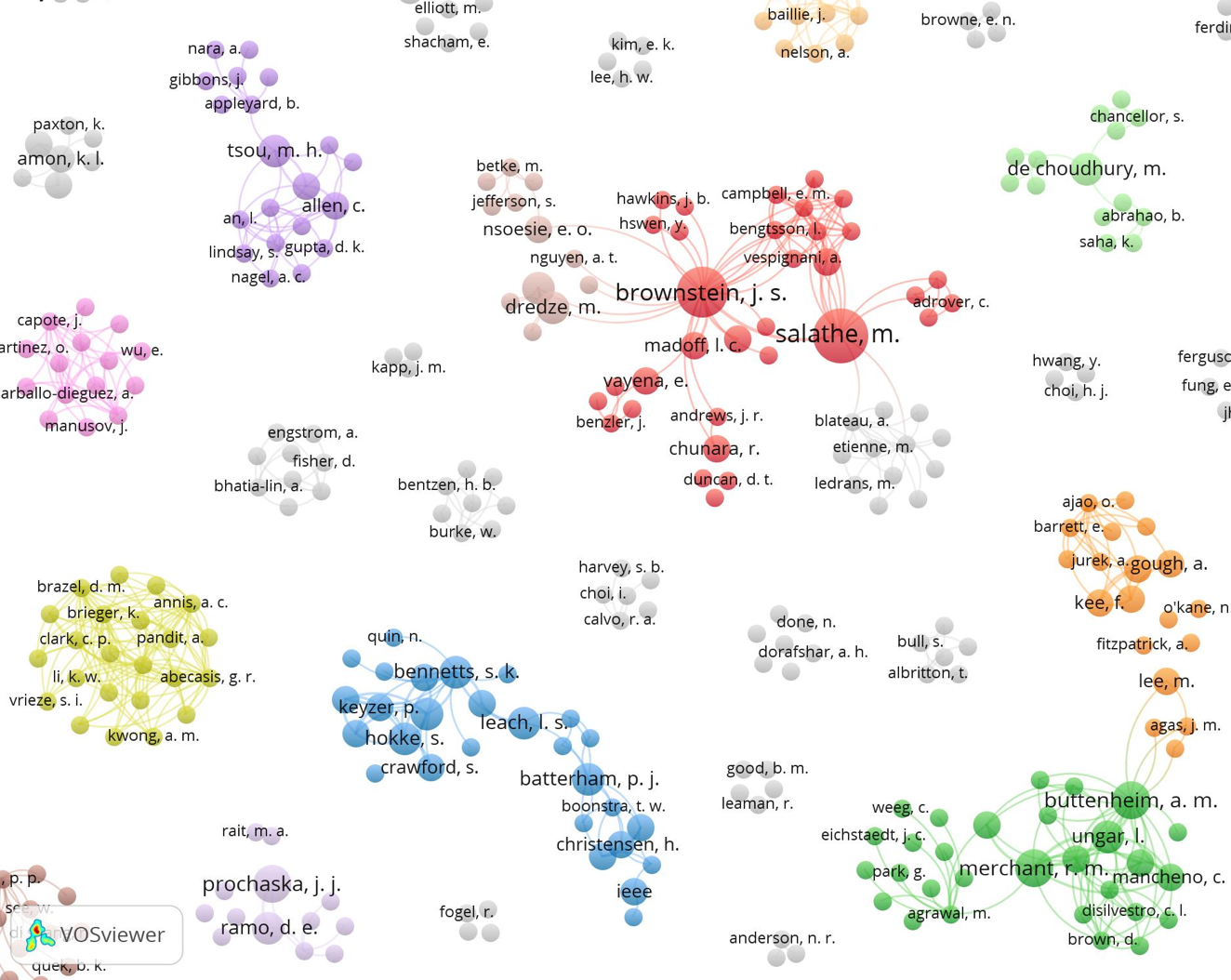

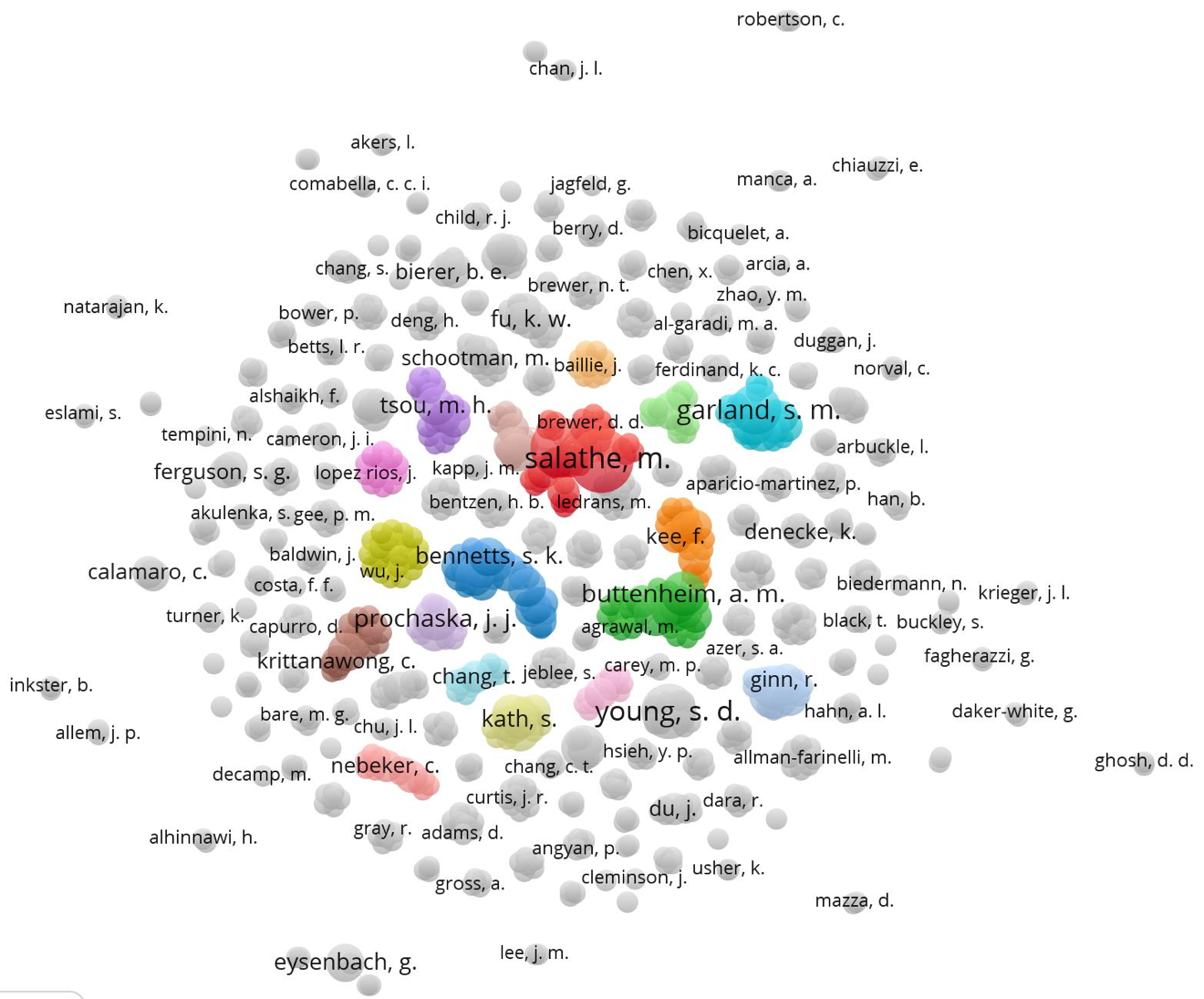


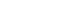


**Figure S1. Coauthorship network of the 1025 authors.**


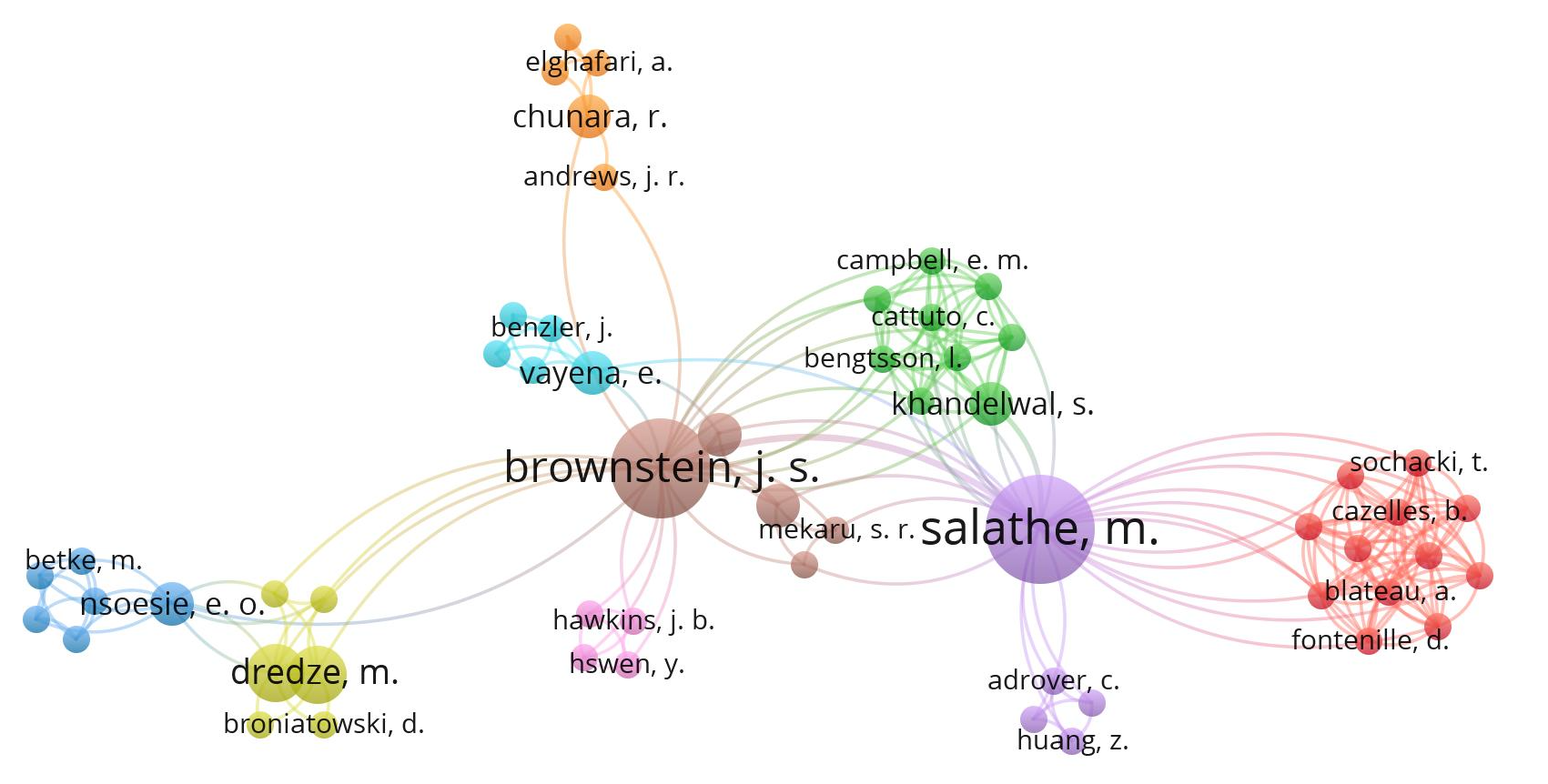


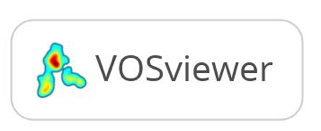


**Figure S2. Largest set of connected authors.**

Supplement: Multimedia Appendix 2 [file jmir_v23i5e25736_app2.docx]
